# Beyond Reported Rates: A Critical Narrative Review of the Risk Architecture, Early Recognition, and Prevention of Complications in Gynecologic Laparoscopy

**DOI:** 10.3390/clinpract16100176

**Published:** 2026-09-22

**Authors:** Rastko Maglic, Milica Ivanovic, Ksenija Lalic, Dragana Maglic, Jelena Stulic, Stefan Ivanovic

**Affiliations:** 1 Obstetrics and Gynecology Clinic “NarodniFront”, 11000 Belgrade, Serbia; 2 Faculty of Medicine, University of Belgrade, 11000 Belgrade, Serbia

**Keywords:** gynecologic laparoscopy, surgical complications, risk factors, early recognition, postoperative red flags, prevention, bowel injury, urinary tract injury, hemorrhage, narrative review

## Abstract

Reported complication rates in gynecologic laparoscopy are widely used as indicators of safety, yet they often describe fundamentally different clinical realities. Variations in definitions, severity classification, duration of follow-up, and procedural context mean that superficially similar outcomes are frequently not comparable. As a result, the literature is rich in data, but limited in interpretability. This review addresses that gap by critically examining complications not only as events but as phenomena shaped by methodological heterogeneity, procedural complexity, and the conditions under which they are recognized. A search of PubMed/MEDLINE, Scopus, and Web of Science was performed, with additional manual reference screening. Given the predominance of heterogeneous retrospective series, cohort studies, registries, and targeted systematic reviews, a narrative synthesis was undertaken. Across studies, the most clinically significant complications remain hemorrhage, bowel injury, and urinary tract injury, while pneumoperitoneum-related events, vaginal cuff dehiscence, port-site complications, port-site metastasis, and morcellation-related dissemination represent rarer but disproportionately consequential outcomes. Importantly, complication risk consistently clusters around identifiable domains, including prior surgery and adhesions, procedural complexity, energy use, operative duration, surgeon experience, and center-level standardization. This review shows that complications in gynecologic laparoscopy cannot be meaningfully interpreted through incidence alone. Their true clinical significance lies in the interaction between risk architecture, intraoperative decision-making, and the timeliness of postoperative recognition. The principal contribution of this work is therefore not a single summary rate but a clinically oriented framework that integrates interpretation of the literature with practical principles of prevention and early diagnosis.

## 1. Introduction

Laparoscopic surgery in contemporary gynecology has long ceased to be merely a less invasive alternative to the open surgical approach. Laparoscopy has become the standard for the treatment of a large number of both benign and, in certain situations, malignant diseases, precisely because it enables less operative trauma, faster recovery, and shorter hospital stay [1,2,3]. However, the increasingly frequent use of laparoscopy in practice has not simultaneously simplified the issue of its safety; on the contrary. The expansion of the spectrum of indications, increasingly complex procedures, and surgery in patients with a history of previous abdominal interventions, adhesions, a larger uterus, or significant comorbidities have led to the fact that complications today represent one of the key issues in minimally invasive gynecologic surgery, rather than a secondary problem [4,5,6,7,8,9].

The clinical significance of complications in gynecologic laparoscopy does not arise from their frequency but primarily from their diversity, severity, and the consequences they may have for the further course of treatment, that is, for the patient herself [10,11]. Within this same framework are bleeding, injuries to the bowel, urinary bladder, ureter, and major blood vessels, but also rarer events with disproportionately high clinical significance, such as complications associated with pneumoperitoneum, vaginal cuff dehiscence, port-site hernias, port-site metastases, and dissemination of unrecognized malignancy during morcellation [11,12,13,14]. What these events share is not only the possibility of leading to serious morbidity, reintervention, or conversion but also the fact that their outcome often depends on the moment of recognition. Precisely for this reason, the safety of the laparoscopic approach cannot and must not be assessed solely through the overall complication rate [9,15]. Such an approach assigns equal value to clinically less significant events and to those which, although rarer, may determine the entire postoperative course and substantially diminish the previously mentioned advantages of minimally invasive surgery [10,11,16].

Despite the large number of published studies and registries, the available literature does not provide a completely clear and readily comparable picture of the true burden of complications. Differences among studies do not relate only to the type of procedures, operator experience, and population characteristics but also to the very definitions of complications, their classification, and the duration of postoperative follow-up. While one group of authors distinguishes between intraoperative and postoperative complications, certain authors use a division into major and minor complications: in some studies, the Clavien–Dindo classification is applied; Kumakiri et al. record as complications exclusively intraoperative organ injuries requiring repair; and some authors combine the aforementioned classifications [12,15,17,18,19,20,21,22,23]. At the same time, studies limited exclusively to the period of hospitalization consequently overlook complications that manifest after discharge [20]. Therefore, the problem in this field is not the lack of published data, but the lack of a sufficiently coherent and critically structured synthesis.

For these reasons, this paper was conceived as a critical narrative review of major and rarer but clinically consequential complications of gynecologic laparoscopy. Previous reviews have primarily addressed specific organ-system injuries, life-threatening events, port-site complications, broad descriptive overviews of laparoscopic complications, or practical aspects of intraoperative prevention and management [10,11,14,15,16]. The distinctive contribution of the present review is to integrate these domains within a single clinically oriented and methodologically critical framework. Rather than treating reported complication rates as directly comparable measures of safety, we examine how differences in definitions, severity classification, duration of follow-up, procedural complexity, and timing of recognition shape the observed burden of morbidity and relate these methodological limitations to recurrent risk patterns, prevention, early recognition, and system-level quality improvement. Accordingly, the aim is not to provide another catalogue of adverse events or a single summary complication rate but to clarify how heterogeneous evidence can be interpreted and translated into clinically meaningful risk assessment and safer laparoscopic practice.

## 2. Methodology

### 2.1. Review Design

This paper is a critical narrative review of the literature on the major and rarer, but clinically significant, complications of gynecologic laparoscopy, with particular emphasis on their incidence, risk factors, mechanisms of occurrence, early recognition, prevention, and the limitations of the available evidence. The narrative approach was selected because of the marked heterogeneity of the available literature, which includes retrospective single-center and multicenter series, registries, cohort studies, systematic reviews addressing individual complications, and a smaller number of papers focused on rare but clinically significant events. Formal quantitative pooling of heterogeneous data could create an impression of methodological precision without true clinical comparability. Although elements of a structured literature search and selection were applied, including a PRISMA-like flow diagram of study inclusion, they were used exclusively for the sake of transparency and do not imply that this paper was conducted as a systematic or scoping review.

### 2.2. Literature Search Strategy

The literature search was conducted in the PubMed/MEDLINE, Scopus, and Web of Science databases. The primary search included papers published from January 2005 to March 2026, with full text available and data derived from human studies. Older papers of particular importance, including seminal series, registries, and guidelines, were additionally included through manual review of the references of selected publications. The search strategy was based on combinations of keywords and, where applicable, MeSH terms using the Boolean operators AND/OR. The search included combinations of terms: gynecologic laparoscopy, laparoscopic hysterectomy, surgical complications, bowel injury, urinary tract injury, vascular injury, hemorrhage, port-site complications, vaginal cuff dehiscence, pneumoperitoneum, morcellation, port-site metastasis, risk factors, prevention, and conversion to laparotomy. Additional relevant publications were identified through manual review of the reference lists of the included papers. This approach was chosen because the literature on complications of gynecologic laparoscopy is not organized around a unified terminology but is instead dispersed across papers of different designs and different focuses.

### 2.3. Study Selection

The selection of publications was carried out through several predefined steps. After combining the results from all databases, duplicates were removed, and the titles and abstracts of the papers were then screened to assess their thematic relevance. In the second step, the full texts of potentially eligible studies were analyzed in order to assess their methodological and clinical value for the purpose of this review. Particular attention was given to whether the study clearly defined complications, described their clinical context, allowed interpretation of risk factors or mechanisms of occurrence, and whether it contributed to the understanding of prevention, early recognition, or the limitations of the existing data.

### 2.4. Inclusion and Exclusion Criteria

The review included studies that:

1. analyzed intraoperative or postoperative complications of gynecologic laparoscopy;
2. presented data on incidence, risk factors, mechanisms of occurrence, outcomes, or preventive measures;
3. addressed major complications, such as bleeding, injuries to the bowel, urinary tract, and major blood vessels, or rarer but clinically significant complications such as complications related to pneumoperitoneum, port-site complications, vaginal dehiscence, and morcellation-mediated dissemination;
4. represented guidelines, consensus documents, or systematic reviews relevant to the interpretation and clinical application of the findings.

Studies were excluded if they:

1. focused exclusively on technical aspects of the procedure without a clear relation to complications;
2. presented therapeutic outcomes without sufficient data on complications, their definition, or clinical context;
3. were available only in the form of an abstract, letter to the editor, or comment without sufficient analytical value;
4. were without full-text availability.

Individual case reports were not automatically excluded, but were considered only for rare, clinically consequential complications when higher-level evidence was unavailable and when they provided unique information on mechanism, diagnostic presentation, or prevention. They were treated exclusively as illustrative, hypothesis-generating evidence and were not used to estimate incidence, comparative risk, or the effectiveness of preventive strategies. Whenever available, greater evidentiary weight was assigned to guidelines and consensus documents, systematic reviews, registries, and larger cohort studies. Accordingly, case reports were used only to contextualize exceptional presentations or mechanisms and were explicitly distinguished from higher-level evidence throughout the narrative synthesis.

### 2.5. Data Extraction and Narrative Synthesis

From the included studies, the following domains were qualitatively extracted: type of complication, time of occurrence and time of recognition, mechanism of injury, reported incidence, population characteristics, risk factors, clinical consequences, management approach, preventive measures, and methodological features of the study, including the way complications were defined and the duration of follow-up. The data were then synthesized thematically and narratively, with the aim of identifying patterns that are consistently repeated across different types of studies, as well as areas in which the literature remains methodologically inconsistent or clinically indeterminate. Key studies were prioritized according to their study design, sample size, methodological transparency, clarity of complication definitions and follow-up, relevance to the specific clinical question, and applicability of the findings. Guidelines and consensus documents, systematic reviews, registries, and larger cohort studies were given greater evidentiary weight for incidence estimates and broadly applicable clinical conclusions. Smaller observational studies and individual case reports were used more selectively, primarily when they provided clinically important information on rare complications, mechanisms, or diagnostic presentations not adequately captured by higher-level evidence. Individual case reports were treated exclusively as illustrative, hypothesis-generating evidence and were not used to estimate incidence, comparative risk, or the effectiveness of preventive strategies. A formal meta-analysis was not conducted because of the heterogeneity of complication definitions, differences in populations, type of procedure, level of operator experience, and length of postoperative follow-up, all of which directly limit meaningful numerical comparison of the results.

### 2.6. Methodological Limitations

The main limitation of this paper arises from its narrative design. The available literature on complications of gynecologic laparoscopy is methodologically highly heterogeneous. It includes single-center experiences, registries, retrospective cohorts, and studies that use different definitions of complications, different classification systems, and different follow-up time frames. For this reason, the direct comparability of the findings remains limited. Additionally, part of the literature, particularly on rarer complications, is based on smaller studies and individual case reports, which increases the risk of publication bias and overestimation of unusual clinical presentations. In this paper, a formal assessment of the risk of bias using tools intended for systematic reviews was not performed because such an approach would not be fully appropriate for the heterogeneous nature of the included evidence. Instead, the studies were interpreted critically, taking into account their design, sample size, clarity of definitions, duration of follow-up, and clinical applicability of the findings. Therefore, the conclusions of this review should be regarded as a critically synthesized framework for understanding the problem, rather than as an attempt at a formal quantitative assessment of a single risk rate.

## 3. Discussion

### 3.1. The Problem of Definitions and Classification

Any serious discussion of complications must begin with the fundamental question of what a complication actually entails. This is precisely one of the main reasons for the methodological inconsistency of the available literature. In some studies, complications are divided into intraoperative and postoperative; in others, into major and minor; in some papers, the Clavien–Dindo classification is used for postoperative events; while certain authors consider as a complication exclusively an organ injury requiring immediate repair. There are also papers that, instead of organizing complications according to the time of occurrence or severity, classify them according to the affected organ or the mechanism of injury [12,15,17,18,20,21,22,23]. At the level of clinical reasoning, all of these classifications may be justified. The problem arises when the results of studies defined in this way are placed side by side and begin to be interpreted as if they were referring to the same phenomenon. This is best illustrated by the example of the division into major and minor complications. At first glance, it is intuitive and practical, but it is not always consistently defined. In one study, transfusion will be designated as a major event because it indicates serious bleeding, while in another it will be classified as a lower-grade complication if it did not require reoperation, and in a third it will be part of Clavien–Dindo grade II and thus remain outside the category of “severe” complications in the narrower clinical sense [12,20,24]. The same also applies to hematomas, urinary infections, transient episodes of fever, or minor port-site infections. When such events are included in one group, the overall complication rate increases [15,20]. When they are excluded or recorded only incidentally, it will be lower [17,18,21,25,26]. The number, therefore, is the same only formally, but its meaning is not the same.

The Clavien–Dindo classification represents an attempt to overcome part of this heterogeneity. Its advantage lies in the fact that it does not assess a complication solely according to the type of event but according to the therapeutic treatment that the event required. This gives it a certain degree of objectivity and makes it particularly useful in retrospective studies and registries. When Clavien–Dindo is combined with clear recording of specific intraoperative incidents, primarily injuries to the bowel, urinary tract, and blood vessels, it probably provides the most meaningful framework for presenting overall perioperative morbidity. However, because this system has not been universally adopted in all studies dealing with complications of gynecologic laparoscopy, the problem of non-comparability has not been resolved. Moreover, in numerous studies with a large number of operations, Clavien–Dindo was not applied at all, which further complicates the interpretation of published rates [12,18,27,28,29,30,31,32,33,34].

A particular methodological problem arises when a complication is defined too narrowly. Kumakiri et al., for example, essentially reduced complications to intraoperative organ injuries requiring repair [23]. Such an approach has its rationale when the aim is a precise analysis of surgical safety in a high-risk population, particularly in patients with previous laparotomies. However, it consequently leads to the exclusion of an entire spectrum of postoperative complications that may have serious consequences, even though they are not immediately recognized during surgery. For this reason, studies of this type may report a lower overall complication rate while at the same time including only the most severe and narrowest segment of the problem. This is not, in itself, a methodological error. The error arises only when results obtained in this way are compared with registries or cohorts that record both minor complications and complications diagnosed later.

From all of the above, a simple but important conclusion follows: in the literature on complications of gynecologic laparoscopy, there is no single universally accepted definition of complication that would allow completely direct comparison of all studies. Therefore, every published result must be interpreted in light of the classification system that was used. Without this step, comparison of the numbers becomes methodologically superficial and clinically unreliable.

### 3.2. The Problem of Different Follow-Up Durations

If the problem of definition is the first major obstacle in interpreting the literature, the duration of follow-up is the next. Complications of gynecologic laparoscopy do not all occur at the same time. While vascular injury during entry, organ perforation during dissection, and uncontrolled intraoperative bleeding are immediate and almost instantly recognizable complications, other complications are detected only hours or days later, when pain, distension, fever, leukocytosis, hemoperitoneum, or signs of sepsis develop. A third category of complications becomes clinically apparent only after discharge; this group includes port-site hernias, vaginal cuff dehiscence, urinary fistulas detected later, or delayed recognition of thermal injuries. From this simple temporal fact follows an important methodological consequence: a study that follows patients only until hospital discharge will never capture the same extent of complications as a study with active 30-day or 6-week follow-up [12,16,17,18,21,25,26,33]. Salmanov et al. followed patients for 30 days and explicitly stated that 74.9% of complications were detected only after discharge [20]. Kaya et al. used a 6-week time window and included all complications that occurred during that period, together with the Clavien–Dindo classification of postoperative outcomes [12]. Kumakiri et al. also recorded a significant number of complications diagnosed postoperatively, and not only during hospitalization itself [23]. The common message of these studies is clear. A longer follow-up window does not increase the number of complications but rather reveals what shorter surveillance would inevitably miss. This is especially important for complications that tend to have delayed manifestation. Thermal injuries of the bowel or ureter may not be recognized immediately. Smaller hematomas or port-site infections may also become clinically relevant only after the patient has gone home. With a shortened hospital stay, which is otherwise one of the main characteristics of the minimally invasive approach, the likelihood paradoxically increases that some complications will not present in the hospital but rather during the period of postoperative outpatient follow-up. If that period is not actively monitored, the study automatically underestimates the true burden of postoperative morbidity. Therefore, a low complication rate in studies with short follow-up does not necessarily mean that outcomes are truly better; it often only means that the follow-up period was shorter [12,14,34,35,36,37].

It should also be kept in mind that different follow-up durations affect not only the overall number of complications but also their profile. Shorter follow-up favors intraoperative incidents and severe early postoperative complications requiring urgent intervention. Longer follow-up introduces an entire spectrum of complications, such as urinary infections, febrile episodes, smaller hematomas, port-site infections, delayed perforations, and similar events that may prolong recovery and increase the number of necessary follow-up visits and treatment costs, even though not all of them require reoperation [10,12,15,17,20,26,37]. Therefore, two studies with a similar surgical population, but a different surveillance window, may yield very different complication rates without any real difference in the technical quality of the operations. Therefore, when an author reports the overall complication rate, it is necessary to verify for how long the patients were followed up and whether there was active post-discharge surveillance. Without that information, the overall complication rate remains insufficiently interpretable.

### 3.3. Why Published Complication Rates Are Not Directly Comparable

When differences in the definition of complications are combined with differences in the duration of follow-up, it becomes clear why published complication rates in gynecologic laparoscopy vary so greatly. However, there are also differences in the population itself, the type of procedure, the experience of the operator, and the level of the center. It is not the same when a study includes diagnostic laparoscopies, minor adnexal procedures, and sterilizations or analyzes total laparoscopic hysterectomies, extensive myomectomies, procedures for deep endometriosis, or operations performed in the presence of massive adhesions. In the first case, the expected risk is lower; in the second, it is, as a rule, higher [9,15,38,39,40,41,42]. If these populations are uncritically grouped together under the term “gynecologic laparoscopy,” the result is an appearance of comparability that does not reflect the true burden of complications.

Intraoperative complications in the analyzed series range from very low values, around 0.32%, to higher rates reaching 5.6%. Postoperative complications in many single-center studies amount to approximately 2% to 6.5%, whereas in Salmanov’s Ukrainian, multicenter registry, the overall complication rate reached 16.2%, of which 5.04% were major and 11.16% minor complications. At first glance, such results may create the impression of a dramatic difference in quality. However, closer reading shows that large registries capture a broader spectrum of events, include centers with different levels of experience, and record more complications precisely because they search for them more actively and define them more broadly. In contrast, single-center series from high-volume institutions often report lower figures, but at the same time, they not infrequently focus on perioperative or more clinically significant complications, while some milder and delayed events remain outside the registry [9,10,12,15,20,32]. In this sense, a higher reported complication rate does not necessarily indicate poorer surgical quality but sometimes reflects a more transparent recording system [43,44]. Conversely, a very low complication rate does not automatically imply outstanding surgical quality, but may reflect patient selection, the experience of an individual expert, a narrower scope of definitions, or a limited surveillance period. This is especially true for studies by a single operator or a single center, which are of great value when they present technically standardized work, but whose results are not applicable to hospitals with lower capacity, more heterogeneous teams, and a different patient structure.

An additional problem is the fact that even within the same type of operation, complications are not evenly distributed. Hysterectomies for large uteri, operations after previous laparotomies, procedures involving extensive adhesiolysis, and operations for malignant indications objectively carry a higher risk of bleeding, organ injury, and conversion. If a study does not stratify the results according to procedural complexity, its overall complication rate remains a crude average value that may conceal important clinical differences. Therefore, it is often more useful to consider the pattern of complications and their determinants than the absolute percentage as an isolated figure [4,7,12,22,23,39].

For all these reasons, published complication rates in gynecologic laparoscopy should be interpreted as contextual rather than universal indicators of risk. Their true value is not in serving for simple ranking of studies or centers but in helping to understand patterns of morbidity, namely, which complications predominate in certain procedures, in which populations complications are more frequent, when they are most commonly detected, and under which conditions they remain underestimated. In this way, the literature becomes clinically useful, not because it provides one “true” complication rate but because it shows why such a single rate probably does not even exist. This heterogeneity is further illustrated by a comparison of key studies summarized in Table 1.

## 4. Major Complications

The key clinical characteristics of major complications, including their mechanisms, early warning signs, and preventive strategies, are summarized in Table 2.

### 4.1. Hemorrhage and Major Vascular Injury

Hemorrhage is one of the most important complications of gynecologic laparoscopy because it directly alters the course of surgery; affects the need for transfusion, reintervention, or conversion; and rapidly eliminates the advantages of the minimally invasive approach if it is not brought under control in a timely manner. Its reported incidence in the literature depends on the type of procedure and the way in which the complication is defined. In general studies of gynecologic laparoscopic surgery, transfusions are mostly rare, whereas they are considerably more frequent in cohorts including hysterectomies, large uteri, myomectomies, or oncologic indications. In other words, hemorrhagic risk is not a general property of laparoscopy but rather relates to the specific procedure, the patient’s anatomy, and the population in which it is performed [7,12,22,45,46,47].

The sources of bleeding most commonly belong to one of three groups. The first consists of entry injuries during placement of the Veress needle and trocars, which may lead to direct damage to major blood vessels or vascularized structures of the anterior abdominal wall [16,48]. The second group consists of bleeding occurring during dissection, especially in the presence of adhesions, bulky myomas, altered anatomy, or extensive retroperitoneal dissection [20,49,50,51]. The third group includes postoperatively manifest bleeding after apparently satisfactory intraoperative hemostasis. The latter category often represents the greatest practical problem because hemostasis at the end of surgery may appear adequate, while in the early postoperative hours, hemoperitoneum, a drop in hemoglobin, and hemodynamic instability develop [12,52].

Risk factors for significant bleeding that recur fairly consistently across studies are procedural complexity, previous abdominal operations and adhesions, a large uterus or large myomas, longer operative duration, preoperative anemia, and lack of operator experience. During surgery, it is particularly important to distinguish between a technically difficult but controlled operative course and a situation in which visualization has already deteriorated to the extent that further laparoscopic continuation of the procedure no longer increases safety but rather risk. Hence, conversion has a special place in hemorrhagic complications. Thus, when timely, conversion is not a sign of failure but a therapeutic measure aimed at establishing control over the further operative course [39,53,54,55,56].

Major vascular injuries are rare but, clinically, extremely important. In most studies, their incidence remains below 0.3%. Salmanov et al. recorded 13 major blood vessel injuries during insertion of the Veress needle, corresponding to a rate of 0.09%. These figures appear low only until they are translated into clinical reality, which includes massive bleeding, emergency laparotomy, vascular reconstruction, intensive care treatment, and a potentially fatal outcome. In such injuries, there is no room for hesitation. The most important question is often not how to stop the bleeding laparoscopically but when the moment has come to proceed urgently to an open approach [15,20,27,49,57].

Clinical recognition of a hemorrhagic complication requires a low threshold of suspicion. Intraoperatively, it is prompted by active bleeding, worsening visibility, a drop in blood pressure, tachycardia, and inability to maintain a dry field. Postoperatively, the picture is less obvious, but no less serious. Thus, symptoms and signs such as progressive abdominal pain, tachycardia, hypotension, syncope, distension, and an unexpected drop in hemoglobin must not be attributed to “slower recovery”. In this context, laboratory assessment, ultrasound, and CT or CT angiography are not supplementary methods, but part of the basic algorithm. The outcome depends to the greatest extent on the speed of diagnosis and the effectiveness of hemostasis [11,12,15,52,53].

Prevention begins before surgery and does not end with the last hemostatic suture. Correction of anemia, risk assessment in the setting of a large uterus and previous operations, careful planning of the approach, and realistic anticipation of difficult dissection reduce the likelihood of serious hemorrhage. Intraoperatively, safe entry, disciplined hemostasis, continuous assessment of whether the operative field remains safe for continuation of laparoscopy, and a final inspection that must be active rather than merely formal are crucial. In hemorrhagic complications, the quality of the surgeon is demonstrated not only by skill in dissection but also by the ability to recognize in time the limit beyond which insistence on laparoscopy no longer protects the patient [15,16,52,58,59].

### 4.2. Bowel Injuries

Bowel injury is one of the most serious complications of gynecologic laparoscopy, not because it is the most frequent but because a missed diagnosis can be exceptionally dangerous for the patient. In the available literature, the reported incidence ranges from approximately 0.09% to 11.4%, with the highest values originating from selected high-risk cohorts, particularly in patients with previous laparotomies and existing adhesions. The wide range in the incidence of bowel injuries itself shows that bowel injury is not a single event but rather a collection of injuries occurring under different clinical circumstances and with very different outcomes [12,15,21,23,31].

Three mechanisms dominate across almost all studies. The first is entry into the abdomen, when the Veress needle or trocar may directly perforate a bowel loop, especially if adhesions are present between the anterior abdominal wall and the bowel [60]. The second is adhesiolysis, in which normal anatomic planes are lost and the bowel becomes part of a scarred block that is difficult to separate without tension or mechanical trauma [23,34,61]. The third is the use of energy. Thermal lesions are precisely the most troublesome because they may not be morphologically obvious immediately, and their consequences become visible only after the development of necrosis and leakage of intestinal contents [35,62]. The clinical severity of bowel injury is determined by its location and mechanism of occurrence.

A clinically significant distinction exists between bowel injuries recognized during surgery and those detected postoperatively. A mechanical perforation that is seen immediately and repaired appropriately often has a good prognosis. If there is no severe contamination, laparoscopic repair may be sufficient. The opposite scenario is a delayed-diagnosed injury, particularly a thermal one. In that case, the postoperative course may initially appear nonspecific, and then progressive pain, distension, leukocytosis, fever, peritonitis, sepsis, and, ultimately, the need for more extensive bowel resection develop. Every missed hour increases the consequences of a lesion that may initially have been small [16,34,63].

Clearly recognizable risk factors are previous operations, dense adhesions, extensive adhesiolysis, deep endometriosis, complex oncologic procedures, and uncontrolled or overly aggressive use of energy. Under these conditions, the problem is not only technical complexity but also a more rapid loss of safe anatomic planes. When the operator is no longer working within recognizable anatomy, every subsequent movement carries a greater risk of leading to a lesion that may not be noticed immediately. Therefore, in bowel injuries, technical skill is just as important as the judgment of when the procedure should be slowed down, when the operative plan should be changed, or when conversion to an open approach is necessary [10,16,23,34,56,64].

Postoperative recognition of bowel injury requires discipline and a high degree of clinical suspicion. Pain that worsens rather than subsides, distension, unexplained tachycardia, leukocytosis, elevation of inflammatory markers, and peritoneal signs are not normal postoperative findings after laparoscopy [10,34,35,63]. They represent a deviation that requires investigation. This is precisely where the trap of the minimally invasive approach lies because since the incision is small, there is a temptation to underestimate a serious intra-abdominal complication. Therefore, when suspicion exists, the threshold for further diagnostics must be low.

Prevention of bowel injury begins with the choice of safe entry in patients with likely adhesions, continues with atraumatic adhesiolysis and thoughtful use of energy, and includes careful inspection of adjacent bowel loops that were exposed to difficult dissection or energy application [10,16,34,63,64]. In bowel injuries, the prognosis may be good or poor depending on whether the injury was recognized while it was still without a pronounced systemic response and while it was still manageable [34,63,64,65,66].

### 4.3. Urinary Tract Injury

Injuries to the urinary tract are generally less frequent than hemorrhagic complications and bowel injuries, but their clinical significance is greater than their incidence alone suggests [34,67,68,69,70]. Importantly, bladder and ureteral injuries should be considered separately because their frequency, mechanisms, timing of recognition, and clinical consequences differ. In the systematic review by Wong et al., which included 90 studies and 140,444 gynecologic laparoscopic procedures performed for benign indications, the overall incidence of lower urinary tract injury was 0.33% (95% CI, 0.30–0.36). Bladder injury occurred in 0.24% of procedures (95% CI, 0.22–0.27), whereas ureteral injury occurred in 0.08% (95% CI, 0.07–0.10), making bladder injury approximately three times more frequent [70,71,72]. These estimates should not be interpreted as universal rates because the reported incidence varies according to procedure type, case complexity, definitions, and surveillance strategy. Cystotomy is more often recognized during the operation itself and, when managed appropriately, most often has a good outcome. Ureteral injury is less frequent, but from the standpoint of late morbidity, it is much more problematic precisely because it often remains unnoticed until urine leakage into the abdomen, ascites, elevated creatinine, fistulas, or secondary infections occur [17,18,21,23,25,26,65,66,67,70,71,72].

The anatomic mechanism of these injuries is relatively clear. The bladder is at risk during dissection of the lower uterine segment, opening of the vesicouterine space, and in the presence of adhesions, especially after previous cesarean sections or other operations. The ureter is at risk in situations in which surgery is performed along its course, but without its clear identification, particularly during hysterectomy, skeletonization of the uterine vessels, adhesiolysis, and procedures for endometriosis or malignancy. The problem is further complicated by energy use because the ureter does not have to be transected to be seriously injured; lateral thermal spread alone is sufficient for delayed wall necrosis to occur [68,69,70,71,72].

Unlike vascular injury, which is most often recognized immediately, urinary injuries often reveal their complexity only later. In the case of the bladder, intraoperative diagnosis is relatively common, and closure and catheterization generally resolve the problem. In ureteral injuries, the course is more unpredictable. The patient may initially appear stable and then develop pain, fever, ileus, ascites, urine leakage, or impaired renal function. From that moment on, the lesion is no longer merely an accidentally incurred injury in the operating room but a complex reconstructive problem. Therefore, more than in most other complications, the timing of detection is crucial for the outcome. Early diagnosis allows stenting or primary repair under considerably more favorable conditions. Late diagnosis prolongs treatment and increases the risk of permanent dysfunction [67,73,74,75].

Risk factors that recur consistently across most studies are laparoscopic hysterectomies, previous operations, adhesions, deep endometriosis, oncologic procedures, and any situation in which the anatomy is distorted. It is important to emphasize that operator experience here does not mean only technical confidence but also the habit of actively identifying the ureter when it is near the operative field. It is not enough to know where the ureter should be located. The ureter must be seen, followed, and protected whenever the operative situation requires it. This is precisely where the difference is made between routine laparoscopy and truly safe laparoscopy [68,73,76,77].

Early postoperative recognition of urinary injury requires that certain signs not be overlooked. Oligoanuria, ascites, elevated creatinine, persistent pain, fever, urine in the drain, fistulous leakage, or unexplained prolonged recovery after a technically demanding operation must prompt targeted investigations. Cystoscopy, retrograde urography, and appropriate radiologic methods are part of the logical diagnostic pathway. In these complications, the greatest error is late suspicion [73,78,79].

Prevention is based on precise anatomic orientation, good visualization of the ureter, ureterolysis when necessary, careful dissection along the uterine vessels, and thoughtful use of energy. In the bladder, respect for the anatomic planes is crucial, especially in the presence of adhesions after cesarean section. In urinary injuries, the problem is not primarily a dramatic intraoperative event, but the possibility that a relatively small intraoperative lesion may remain clinically unrecognized long enough to produce a disproportionately large postoperative burden [16,73,74,75,80].

**Table 2:** Major complications of gynecologic laparoscopy: clinical significance, mechanisms, early warning signs, and prevention.

Complication	Typical Incidence in the Literature	Dominant Mechanisms of Occurrence	Early Warning Signs	Key Risk Factors	Most Important Preventive Measures
Bleeding/hemorrhagic complication	Transfusions are rare in general series and more frequent in hysterectomies and complex procedures	Entry trauma, vascular injury during dissection, inadequate hemostasis, bleeding from the extraction site	Tachycardia, hypotension, drop in Hb, abdominal pain, distension, slow recovery	Complex procedure, large uterus/myomas, adhesions, previous operations, anemia, longer operation	Correction of anemia, planning of entry, careful hemostasis, final inspection, timely conversion
Major vascular injury	Rare, mostly <0.3%	Veress/trocar during entry, retroperitoneal dissection	Sudden hemodynamic instability, profuse bleeding, loss of visibility	Entry into the abdomen under unfavorable conditions, altered anatomy, inexperience, complex retroperitoneal dissection	Safe entry, careful risk assessment, low tolerance for delayed conversion
Bowel injury	Approximately 0.09–11.4%, highest in high-risk cohorts	Trocar/Veress, adhesiolysis, thermal injury, mechanical trauma during manipulation	Progressive pain, distension, leukocytosis, fever, peritoneal signs, sepsis	Previous laparotomies, adhesions, deep endometriosis, complex procedures, aggressive use of energy	Entry planning, atraumatic adhesiolysis, cautious use of energy, inspection of bowel loops at the end of surgery
Urinary bladder injury	Approximately 0.24% in a systematic review of benign gynecologic laparoscopy; varies by procedure and study design	Dissection of the vesicouterine space, previous cesarean section, adhesions, hysterectomy	Urine in the operative field, postoperative pain, leakage, irritative symptoms	Previous operations, adhesions, difficult hysterectomies, altered anatomy	Respect for tissue planes, careful dissection, early intraoperative identification of the lesion
Ureteral injury	Approximately 0.08% in the same systematic review; less frequent than bladder injury, but frequently associated with delayed recognition	Ligation near the ureter, thermal necrosis, dissection along the uterine vessels, endometriosis/adhesions	Oligoanuria, ascites, elevated creatinine, fistula, fever, prolonged ileus/recovery	Hysterectomies, oncologic procedures, adhesions, endometriosis, extensive use of energy	Active identification of the ureter, ureterolysis when indicated, limited use of energy in critical zones

Note: Incidence ranges should be interpreted in light of methodological differences among studies because definitions of complications, duration of follow-up, and types of procedures significantly affect the reported rates. The table is therefore conceived as a clinical–analytical overview, rather than a mechanical comparison of numerical outcomes between studies. The data were synthesized from the studies discussed in this section [7,11,12,15,16,20,21,23,25,26,31,34,35,39,45,46,47,49,50,51,52,53,56,57,58,59,63,64,65,66,67,72,73,74,75,80].

## 5. Uncommon but High-Impact Complications

Complications of gynecologic laparoscopy are most often considered, as previously stated, to include bleeding and bowel and urinary tract injuries, because these groups account for the greatest share of immediate perioperative morbidity. Rarer complications occupy a special place. They occur unexpectedly, may appear to be disproportionately severe relative to the minimally invasive nature of the procedure, and are often more strongly related to technique than to the underlying disease. This group includes complications related to pneumoperitoneum, vaginal cuff dehiscence, port-site complications, and the oncologically most sensitive consequences of tissue manipulation and extraction, including port-site metastases and dissemination after morcellation [9,12,14,16,81,82]. These uncommon but clinically significant complications are summarized in Table 3, with emphasis on their context, impact, and prevention.

### 5.1. Pneumoperitoneum-Related Complications

Pneumoperitoneum is a technical prerequisite for laparoscopybut, at the same time, its most characteristic physiologic burden. While most complications in laparoscopy are associated with dissection, energy, or instruments, here, the problem is built into the very framework of the procedure. Insufflated CO_2_ changes intra-abdominal pressure; affects ventilation, venous return, and hemodynamics; and, under certain circumstances, may also become a source of complications that cannot be reduced to an “ordinary operative incident.” In this sense, complications related to pneumoperitoneum occupy a special place because they connect surgical technique, anesthetic management of the operation, and the patient’s physiologic reserve into a single, highly sensitive system [82,83,84].

In most of the analyzed studies, such events are rare. In the included literature, subcutaneous emphysema occurs in a range from 0.03% to 0.6%, pneumothorax around 0.18%, while cases of gas embolism were not registered in large series, although it is precisely the most dramatic of all complications related to insufflation [12,15,20,85]. These figures should be interpreted cautiously. They do not mean that the physiologic impact of pneumoperitoneum is negligible but rather that clinically manifest complications occur rarely in relation to the enormous number of procedures in which CO_2_ is used routinely.

Subcutaneous emphysema represents the most benign end of that spectrum. It occurs when CO_2_ spreads into the subcutaneous tissue, either because of extraperitoneal insufflation, a loose trocar position, longer operative duration, or high insufflation pressures. Clinically, it is most often recognized by palpable crepitus and swelling of the subcutaneous tissue, first in the anterior abdominal wall and then possibly in higher regions. In most cases, the course is favorable and treatment is conservative, but the importance of this event is not only in its immediate effect. It is a marker that pneumoperitoneum is no longer confined to the space controlled by the operator. In this way, subcutaneous emphysema becomes a warning of a possible problem in access, pressures, or duration of the procedure, rather than merely an incidental postoperative finding [15,20,34,85].

Pneumothorax is a different phenomenon. It does not represent merely the spread of gas outside the abdominal cavity but the entry of gas into an anatomic space in which even a small amount may produce serious respiratory and hemodynamic consequences. In gynecologic laparoscopy, it may arise through direct passage of gas through a diaphragmatic defect, lateral dissection, movement of gas along fascial and perivascular planes, or as a consequence of a pre-existing but previously unrecognized diaphragmatic weakness. Its clinical presentation is often sudden. There is a drop in oxygen saturation, an increase in inspiratory pressures, difficult ventilation, and a change in the auscultatory findings. Unlike many other laparoscopic complications, the problem here does not develop insidiously. It imposes itself immediately. Therefore, in pneumothorax, rapid differentiation between a transient respiratory disturbance and an event requiring urgent drainage, and even interruption or modification of the operation, is crucial [15,85].

Gas embolism represents the rarest, but also the most dramatic, complication of this group. It may occur during insufflation, especially if the needle or trocar injures a blood vessel or if pressurized gas comes into direct contact with an open venous channel. The clinical picture may be fulminant, with sudden hypotension, hypoxia, cardiovascular instability, and a drop in end-tidal CO_2_. There is no room here for observation. The response must be prompt and well rehearsed. It is necessary to stop insufflation, deinsufflate the abdomen, administer 100% oxygen, appropriately position the patient, and provide aggressive hemodynamic support. Precisely for this reason, gas embolism, although rare, is nevertheless important for a review of this type. Recognition and appropriate response in the event of this complication are of inestimable importance [15,85].

What makes this spectrum of complications particularly interesting is the fact that prevention requires coordination. Carefully planned insufflation pressures, good trocar positioning, controlled duration of the procedure, careful monitoring of ventilation parameters, and additional caution in patients with cardiopulmonary comorbidities are required. In other words, complications related to pneumoperitoneum remind us that safe laparoscopy is not only precise dissection but also controlled and adapted technical conditions required by the procedure itself [15,20,34,83,84,85].

### 5.2. Vaginal Cuff Dehiscence

Vaginal cuff dehiscence occupies a special place among rare complications because it represents an almost illustrative example of a complication specifically related to the technique of laparoscopic hysterectomy. It is not a consequence of entry, it is not a consequence of dissection in the classical sense, and it is not a complication that can be explained solely by the overall complexity of the procedure. Here, the problem arises at the site where the operation is formally completed. It depends on the quality of colpotomy, the tissue integrity of the cuff edges, and the method of closure [86,87,88,89]. Doğan et al. report approximately 0.2% dehiscence, while Kaya et al. found approximately 0.4% of cases requiring operative management. However, the clinical severity of this complication far exceeds its incidence because it may lead to evisceration, most commonly of bowel loops, and almost always requires urgent surgical intervention. This is precisely the type of event that strongly compromises the perception of minimally invasive procedures. Small incisions and rapid initial recovery become irrelevant when the patient returns with acute, potentially dramatic late failure of the vaginal closure site [12,22,90]. Pathophysiologically, dehiscence is a combination of mechanical and biological failure. The mechanical component refers to the method of colpotomy and the quality of closure. Excessive thermal trauma to the cuff edges may impair tissue viability and hinder healing, even when the closure formally appears satisfactory. The biological component includes infection, hematoma, poor tissue healing, and premature exposure to stress, including premature sexual activity [86,91,92,93]. The risk is not evenly distributed among all hysterectomies. It increases when the colpotomy is wide, when the cuff edges are excessively thermally damaged, when closure is technically suboptimal, or when local factors that impair healing are present, such as infection or hematoma. Interestingly, the literature is increasingly pointing to the role of a standardized closure technique, including the use of bidirectional barbed sutures, although these data currently derive predominantly from retrospective single-center series [15,22,26,86,88,92,93,94,95,96].

The clinical presentation of dehiscence is often sufficiently striking that it does not go unnoticed; it is accompanied by pain, vaginal bleeding, a sensation of pressure, passage of contents through the vagina, or evisceration. This is a complication whose occurrence is largely preventable. Prevention begins with carefully performed colpotomy, continues with preservation of viable cuff edges without unnecessary thermal damage, and ends with reliable closure. Postoperatively, part of prevention also lies in the quality of patient counseling because, here, the technical success of the operation depends not only on intraoperative work but also on adherence to restrictions during the healing period [86,91,96]. Unlike hemorrhage, where the central issue is control of an acute event, or bowel injury, where delayed diagnosis predominates, vaginal cuff dehiscence represents a complication of the final phase. It demonstrates that laparoscopy does not end with removal of the specimen but with the quality of what remains closed after the operation.

### 5.3. Port-Site Complications

Port-site complications may be the most easily underestimated complications in the entirety of laparoscopic surgery. Because ports are small, there is an intuitive tendency to perceive the problems associated with them as small as well. In a large number of cases, this is indeed true. Wound infections, smaller hematomas, and local pain most often have a limited course and do not drastically alter the overall outcome. However, precisely this apparent benignity conceals the fact that the port is the site at which three important dimensions of safety intersect, namely, the integrity of the abdominal wall, local asepsis, and tissue extraction. When one of these three points is compromised, a disproportionately large problem may arise [14,97].

Port-site complications are not a homogeneous group. In practice, they include local infections, hematomas, and chronic pain, but the most important clinical entity in this group is port-site hernia. The incidence range of these complications is from 0.03% to 0.6%, with a clear emphasis that this is a typical late complication, especially in ports larger than 10 mm, in obese patients, and during longer operations. Thus, port-site hernia is the result of a combination of mechanical stress, quality of closure, and the size of the fascial defect [12,14,15,20,22,37,97].

Port-site hernia has particular significance because it is at the same time both a local and a systemically important complication. Local because it arises from insufficient closure or excessive enlargement of the incision. Systemic because it may lead to bowel incarceration, systemic consequences, and emergency surgery. This complication, which arises during the seemingly least dangerous part of the procedure, may lead to serious late morbidity [14,37,98,99]. Precisely for this reason, port-site hernia must not be reduced to a single sentence about a “rare complication” but must be recognized as an indicator of technical discipline at the completion of the procedure.

Port-site infections and hematomas, although generally less dramatic, are not without clinical significance. They prolong recovery, increase the number of follow-up visits, worsen the patient’s experience, and, in certain cases, represent the starting point for a more serious infection or abscess. In other words, these are complications that may not fall into the same category as vascular trauma or bowel injury, but directly affect the real quality of surgical care. Therefore, they should not be exaggerated, but neither should they be underestimated simply because they rarely require reoperation [14,97,100].

The risk of port-site complications is higher when the port diameter is larger, when the duration of the operation is longer, when the patient is obese, when tissues are manipulated roughly during specimen extraction, and when the layers are inadequately closed. It is particularly important that port-site hernias often occur in such a way that a port that was initially of appropriate size is subsequently enlarged to facilitate extraction, and the fascial defect is then not adequately reconstructed [14,100].

Prevention of this group of complications includes fascial closure for ports of 10 mm and larger, careful closure of the subcutaneous and skin layers, avoidance of unnecessary tearing of the incision during extraction, and consistent asepsis. Here, technological progress does not offer much if basic discipline and respect for fundamental principles fail. This group of complications most clearly demonstrates the importance of the quality of procedural execution even in steps whose significance can easily be underestimated, and precisely in that lies the value of their monitoring [12,14,15,100].

### 5.4. Port-Site Metastasis and Morcellation-Related Dissemination

If the previous complications test the physiologic, technical, and reconstructive safety of laparoscopy, then port-site metastases and morcellation-related dissemination test its oncologic discipline. This is why these phenomena, although rare, must have a special place in any serious review of the literature. Here, the problem is not only a complication of the procedure but also a potentially altered biological course of the underlying disease as a consequence of a failure in operative strategy.

Port-site metastases are now rare, but not negligible. Their incidence in larger studies and reviews is generally below 2%, and in some studies below 0.5%, with a higher frequency in biologically aggressive tumors, peritoneal carcinomatosis, and ascites. This finding is important precisely because it brings the issue back from the domain of abstract fear into the domain of real, but selective, risk. These metastases are the result of an interaction between tumor biology and technical circumstances such as manipulation of tumor tissue, aerosolization, gas leakage, and local contamination of the ports. Therefore, they cannot be discussed separately from the indication and the oncologic context of the procedure [81,101,102,103].

The clinical significance of these metastases does not lie only in local recurrence. Their presence often reflects either the aggressiveness of the underlying disease or a technical failure to respect the malignant nature of the disease. When these complications develop, the question arises as to whether the minimally invasive approach in the specific case was performed appropriately and indicated appropriately. It is precisely for this reason that port-site metastases have disproportionate importance in the discussion of laparoscopic safety [81,101,103,104].

Morcellation-related dissemination is a different, but related, problem. Here, the issue is not necessarily implantation of tumor at the port site but intraperitoneal scattering of tissue, which may have serious consequences if the underlying pathologic lesion is an unrecognized malignancy. Intraperitoneal morcellation may lead to dissemination of an undetected sarcomatous lesion or other malignancies, with poor oncologic outcome. Precisely for this reason, the principle of oncologic safety is established as a priority when deciding on further operative management. Rapid extraction and a smaller incision mean little if the price is dissemination of malignant disease throughout the peritoneum [7,105,106,107,108,109].

It is important, however, not to reduce the problem of morcellation only to malignancies because even in benign lesions, tissue scattering may lead to implantation of lesions in the surrounding area and later clinical problems. Nevertheless, the greatest clinical and ethical burden lies in the oncologic risk. Contained tissue extraction and careful patient selection may reduce uncontrolled tissue dissemination in appropriately selected patients. When malignancy is known or reasonably suspected, morcellation should be avoided [110,111,112,113].

**Table 3:** Uncommon but high-impact complications of gynecologic laparoscopy: clinical significance, dominant context, and key prevention.

Complication	Typical Incidence/Range	Dominant Clinical Context	Why It Is High-Impact	Key Preventive Measures
Subcutaneous emphysema	Approximately 0.03–0.6%	CO_2_ insufflation, longer operations, higher pressures, extraperitoneal insufflation	A marker that gas is escaping the controlled space; may accompany a more serious pneumoperitoneum-related problem	Controlled insufflation pressures, stable trocar position, careful monitoring of ventilation parameters
Pneumothorax related to pneumoperitoneum	Approximately 0.18% in the available series	Diaphragmatic defect, lateral dissection, movement of gas along fascial planes	Abruptly compromises ventilation and hemodynamics; may require urgent drainage and modification of the procedure	Caution during dissection, monitoring of PIP and SpO_2_, rapid intraoperative response
Gas embolism	Exceptionally rare; often no recorded cases in large series	Insufflation in the setting of vascular injury or contact with an open venous lumen	The most dramatic complication of pneumoperitoneum; may be fulminant and fatal	Proper entry, controlled insufflation, an immediate algorithm for emergency management
Vaginal cuff dehiscence	Approximately 0.2–0.4% in the cited hysterectomy series	Laparoscopic hysterectomy, thermally damaged cuff edges, suboptimal closure	May lead to evisceration and urgent reoperation; a test of final reconstructive discipline	Careful colpotomy, viable cuff edges, standardized cuff closure, adequate patient counseling
Port-site hernia	Approximately 0.03–0.6%	Larger ports, obesity, long operations, enlargement of the incision during extraction	A late complication that may result in bowel incarceration and emergency surgery	Consideration of fascial closure for larger or enlarged trocar sites, careful layer closure, and avoidance of rough enlargement of the incision
Port-site infection/hematoma	Low rates; more often minor than major events	Local trauma, extraction, insufficient asepsis	Prolongs recovery and increases the true local morbidity of laparoscopy	Consistent asepsis, careful handling of the port and specimen, good local hemostasis
Port-site metastasis	Generally <2% and in some series <0.5%	Oncologic procedures, aggressive tumors, ascites, carcinomatosis	A local event with systemic oncologic significance; calls into question the oncologic safety of the procedure	ENDOBAG, atraumatic manipulation, prevention of gas leakage and port contamination
Morcellation-related dissemination	Rare, but clinically most important when unrecognized malignancy is present	Hysterectomy/myomectomy with intraperitoneal morcellation	May alter the oncologic course of disease and worsen the outcome	Strict patient selection, avoidance of morcellation when malignancy is suspected, contained morcellation

Note: These complications are important because they indicate the “vulnerable points” of laparoscopic surgery. Therefore, even when they are rare, they should not be neglected, but should always be regarded as an important part of the overall assessment of the safety of the minimally invasive approach. The data were synthesized from the studies and reviews discussed in this section [9,12,14,15,16,20,22,26,34,81,82,83,84,85,86,87,88,89,90,91,92,93,97,98,99,100,101,102,103,104,105,106,107,108,109,110,111,112,113].

## 6. Risk Factors Across Complication Types

After considering the major and rarer, but clinically significant, complications, the following question arises: Does each of them occur as an isolated event related to a specific technical error, or is there a recognizable pattern of risk that links them? Despite its methodological heterogeneity, the available literature fairly consistently supports the latter interpretation. Complications of gynecologic laparoscopy are not distributed randomly. They cluster around several stable characteristics, namely, the patient’s anatomy and previous surgical history, the complexity and structure of the procedure itself, and the experience and organizational capacity of the operating team [12,15,20,23,31,114]. Precisely for this reason, preoperative risk assessment in laparoscopy must not be reduced to routine collection of patient data and the formal statement that a more complex procedure is required. It is necessary to understand how individual factors reinforce one another and how to recognize circumstances in which complications are more likely.

Risk does not mean that a complication will necessarily occur but rather that the operation is entering a zone in which the margin of safety is narrower and the potential outcome less favorable. Some risk factors act directly, for example, the presence of dense adhesions or a large-volume uterus. Others, such as prolonged operative duration, impaired visualization, greater need for energy use, or a higher likelihood of conversion, act indirectly. A third group is not related to the patient or the procedure itself but depends on the level of training, standardization of procedures, the team’s ability to recognize deviations early, and readiness to proceed to conversion when necessary [12,115,116,117]. Only when these three levels are considered together does risk cease to be abstract and become a clinically useful concept. These interacting domains of risk and their practical implications are summarized in Table 4.

### 6.1. Patient-Related Factors

The most consistent patient-related risk factor in the literature is previous abdominal and pelvic surgery, especially when it has left dense adhesions and distorted anatomy. This association is not surprising. In the study by Kumakiri et al., which included patients with prior laparotomies, the rates of intraoperative injuries and conversion were higher than in the general laparoscopic population, and most of the more serious events occurred precisely during adhesiolysis. Larger registries and broader literature reviews show that adhesions increase not only the technical difficulty of the procedure but also the likelihood that a complication will occur at several levels simultaneously. Entry into the abdomen will be more difficult, as will dissection; greater use of energy will be required; the likelihood of bleeding, bowel injury, and ureteral injury will be higher; the operation will last longer; and conversion will be performed more often. Adhesions are therefore a risk multiplier [23,118,119,120].

Another important domain includes the anatomic and morphologic complexity of the patient herself, primarily uterine size, the presence of bulky myomas, and the presence of large masses in the pelvis or abdomen. Such anatomy changes the geometry of the operation. It reduces the working space, prolongs dissection, and complicates specimen extraction. The consequence is not only longer operative duration but also a greater likelihood that the operation will turn from a “routine” procedure into one with multiple sources of tension, such as poorer visualization, greater blood loss, greater need for manipulation, and a rising likelihood of conversion. Louie et al., as well as the studies by Uccella et al., show that increasing uterine weight is accompanied by increasing operative burden and complications [7,115,121,122].

Obesity undoubtedly makes laparoscopy more difficult because access is technically more demanding, visualization is often poorer, the working space is deeper, and the duration of surgery is longer. At the same time, the literature is not entirely unequivocal when the question is whether high BMI is an independent predictor of major complications or merely a marker of a more complex perioperative environment. After adjustment for procedural complexity, adhesions, and comorbidities, BMI itself does not always remain an independent determinant of severe complications, but it is consistently associated with technical difficulties, a higher likelihood of conversion, and greater local morbidity [12,15,20,123,124].

Older age, ASA status, and comorbidities act differently from the previously mentioned factors. They do not change the anatomy itself but rather reduce the patient’s ability to tolerate the operative burden and any eventual complications. A patient with cardiovascular disease, diabetes, a higher ASA status, or pre-existing pulmonary limitation does not enter surgery with the same reserve as a younger and systemically healthier woman. In this sense, the same operative incident will not have the same clinical outcome in two different patients. In older patients with a greater number of comorbidities, the likelihood of infection, thromboembolic events, slower recovery, and prolonged treatment increases. Here, the risk is not related only to the occurrence of a complication but also to the reduced capacity to absorb it without significant consequences [12].

Preoperative anemia and hemostatic disorders should be singled out in particular. They are often treated as an accompanying laboratory finding, whereas in reality they represent one of the most concrete risk factors that can be influenced. Anemia significantly reduces the window for safely tolerating even relatively minor blood loss [45,125,126]. Therefore, the same amount of bleeding does not produce the same clinical effect in two different patients. In this sense, preoperative optimization may contribute to a real reduction in risk.

Taken together, patient-related factors do not act in isolation. Their true weight usually becomes visible only when they “act” together. A patient with previous laparotomies, a large uterus, obesity, and anemia presents a radically different operative situation from a patient without these characteristics. Precisely for this reason, high-quality preoperative assessment must be stratificational and active, rather than merely descriptive and routine.

### 6.2. Procedure-Related Factors

Unlike patient-dependent risks, which determine the circumstances in which surgery is performed, procedure-related risk factors imply the complexity of the procedure itself. Laparoscopic sterilization, a minor adnexal procedure, and total laparoscopic hysterectomy with adhesiolysis, or laparoscopic surgery for deep endometriosis, do not carry the same risk, even though formally they all fall under the category of “gynecologic laparoscopy”. It is precisely the more complex procedures that consistently carry a higher risk of bleeding, bowel injury, urinary tract injury, and conversion. This should not be interpreted only as a consequence of longer duration. A more complex procedure almost by definition means longer time spent in critical anatomic zones, more dissection, more energy use, more frequent need for a change in plan, and, therefore, a greater likelihood that a complication will occur [15,39,127].

Among procedure-related factors, the method of abdominal entry occupies a special place. Although it is the very beginning of the operation, the possibility of serious injury already exists. Precisely for this reason, entry is not a technical formality but the first real test of adequate planning. In patients with suspected adhesions or previous laparotomies, the choice of entry site, as well as the decision between the Veress approach, the open Hasson approach, or an alternative entry site, may be of greater importance than the later details of the dissection itself. A large proportion of serious entry-related injuries occur precisely when the approach becomes routine in situations in which it should not [128,129,130].

The second major procedure-related risk factor is the use of energy. Its importance lies in the fact that it may create a complication that is insufficiently visible at the moment it occurs. A mechanical error is more often seen, whereas a thermal error is more often recognized later. Therefore, the manner and intensity of energy application have broader consequences than may appear at first glance. Thus, energy cannot be viewed only as an instrument for dissection and hemostasis, but also as a potential cause of complications. Especially in conditions of a confined operative field, endometriosis, adhesions, or work near the ureter and bowel, the use and control of energy become a matter of safety [16,62,131,132].

The duration of the operation is another factor that often appears to be a passive consequence of complexity, but in fact carries its own risk. A long procedure means longer exposure to pneumoperitoneum, fatigue of the operator and team, prolonged pressure on tissues, and a greater likelihood of a cumulative effect of small technical compromises. Therefore, duration is not only a descriptive parameter listed in the results but also an independent clinical indicator of how demanding the operation was [12,133].

A distinct procedural domain is constituted by tissue extraction and the final steps of the operation. It is precisely in these steps that some of the most specific vulnerable points of laparoscopy are located (port enlargement, development of hernias, local contamination, quality of vaginal cuff closure, and issues related to morcellation and oncologic safety) [14,134]. In other words, an operation does not become safe merely because the main dissection phase has been completed successfully. The completion of the procedure carries its own risks. In this sense, safe laparoscopy requires discipline and focus both in the more demanding steps and in those that are technically less demanding.

When all procedure-related risk factors are considered together, the message is clear: the more reasons there are for the operator to assess, choose, adjust the strategy, and work under conditions of reduced margin for error, the greater the likelihood that a complication will occur. Thus, procedural risk in laparoscopy is always a risk of cascade. Entry complicated by adhesions may lead to poorer visualization, poor visualization to more difficult dissection, and more difficult dissection may require greater use of energy, which may prolong the duration of the operation and thereby increase physiologic burden and fatigue, and all of this together increases the likelihood that one of the complications will occur.

### 6.3. Surgeon- and Center-Related Factors

The third category of risk factors is the most difficult to standardize, and it refers to operator experience and center capacity. On this, the literature speaks almost unanimously. Single-center studies by experienced operators consistently show lower rates of serious complications, whereas multicenter registries and more heterogeneous cohorts report higher values. This is partly a matter of methodology, but partly also a reflection of the reality that high-volume centers and experienced operators work with a different level of technical routine, anticipation of problems, and ability to stop before the situation escalates [4,15,17,26,27]. Precisely for this reason, experience is not only a factor that reduces errors but one that changes the entire dynamics of the operation. What is particularly important is that experience should not be reduced to the number of procedures performed. Of course, a learning curve exists, and studies report a clear decline in complications after 75–150 procedures, especially with structured training and mentorship. However, operative maturity encompasses more than quantity. It implies the ability to recognize inadequate dissection in time, not to lose orientation in altered anatomy, and to make the decision to convert without psychological resistance when necessary. In this sense, experience brings not only a lower number of complications but also better management when a complication occurs [4,15,26,27].

The capacity and organization of the center operate more broadly than the individual operator. An institution with a high volume of procedures usually has a more standardized perioperative pathway; clearer protocols for entry, adhesiolysis, hemostasis, closure, and prophylaxis; and early diagnosis of complications. It has a team accustomed to the same rhythm of work, anesthesiologists who recognize laparoscopic specificities, more readily available consultants, and a lower threshold for an appropriate response [27]. In other words, a developed center reduces risk not only through technique but also through good organization. This is the reason why two technically similar operations do not necessarily have the same outcome in two different systems. A special, often underestimated, risk factor is the degree of standardization. Complications are reduced not only by experience but also by reduction of variability. When the team uses a standard approach to entry, a clear plan for high-risk patients, consistent final inspection, and a clear algorithm in case of suspected postoperative complication, the probability that a serious problem will develop becomes lower. It is important to understand that standards protect the patient better than improvisation [135,136]. This also brings in the issue of transparency in complication reporting and complication monitoring. A center that does not consistently record minor events, that does not have active follow-up, or in which conversion is perceived to carry a stigma produces a false image of its own outcomes. In contrast, a team that registers complications transparently and uses them for audit and education may report more events, but, at the same time, it will create a more realistic basis for improving practice [11,137]. Finally, surgeon- and institution-related factors also determine the quality of complication management, and not only the quality of prevention [27]. It is not possible to avoid all complications. In this sense, the safest centers are not those in which complications never occur, but those in which a complication rarely remains unrecognized for long and is not poorly managed.

## 7. Early Recognition and Postoperative Red Flags

For a large proportion of complications, the decisive moment does not occur during the procedure itself, but in the hours and days that follow. In most cases, minimally invasive surgery implies an easier postoperative course. However, when complications occur, that initial assumption may delay suspicion, slow diagnosis, and increase the consequences of an event that in its early phase may still have been limited and easier to resolve. Therefore, early recognition of complications belongs not only to the domain of awareness but also to the domain of discipline. Discipline implies readiness to react as soon as the clinical picture begins to deviate [12,16,34].

In this part of the review, we wanted to emphasize the importance of early clinical warning, namely, which signs carry weight; how to distinguish an unpleasant, but still normal, recovery from a process that is becoming pathological; and in what sequence diagnostic tools should be used. The essence is not that every pain, fever, or nausea after laparoscopy should be a reason for panic. The essence is to recognize a deviation from the expected course and not to rationalize that deviation for too long [16,34,138].

### 7.1. When the Postoperative Course Is No Longer Expected

Most patients after uncomplicated laparoscopy have a predictable early course. Pain is present, but tolerable and decreasing; mobilization progresses; hemodynamics are stable; the abdomen is not progressively distended; temperature remains normal or there is only transient low-grade fever; and laboratory changes do not escalate. The problem arises when the expected pattern is disturbed. Clinical practice shows that a complication rarely begins as a striking picture. Much more often, it begins as a not overly conspicuous irregularity that does not follow the rest of the postoperative course: pain that does not subside at the expected rate, tachycardia that does not have a good explanation, distension that increases, hemoglobin that falls more than expected, a temperature that does not seem to be merely a transient inflammatory response, or diuresis that does not correspond to the hemodynamic status. There is no single universal sign of complication; more important is the pattern in which several smaller deviations accumulate into a picture that no longer appears physiologic [10,12,52,122].

Progressive abdominal pain is, in this sense, the most important “red flag”. Pain itself after laparoscopy is not unexpected. However, pain that increases instead of decreases, that changes in character, or that is accompanied by distension or general deterioration ceases to be merely a postoperative complaint and becomes a signal that the cause must be sought. The same applies to abdominal distension. It may be transient and benign in origin, but when associated with pain, nausea, vomiting, delayed recovery, or laboratory changes, it cannot be regarded as a negligible finding. A fall in hemoglobin is the third key sign, especially when it does not fit the expected blood loss and when it is accompanied by tachycardia or hypotension. In such circumstances, the number should not be viewed in isolation but in the context of the clinical picture. A hemodynamically stable patient with a mild fall in hemoglobin is not the same as a patient with a progressive fall in hemoglobin, pain, and inadequate recovery, even if the difference in absolute values is not dramatic [12,20,139].

Elevated temperature occupies a particularly sensitive place in postoperative assessment because it is easy both to overestimate and to underestimate it. An isolated transient fever immediately after the procedure may be of little significance. A temperature that persists, rises, or is associated with local or systemic signs of deterioration requires a different approach. The key question is not whether the patient has an elevated body temperature but whether the clinical picture appears proportional to what was expected from that operation [10,12]. The same applies to oliguria, especially when it cannot be explained by hypovolemia or perioperative circumstances. In combination with pain, elevated creatinine, distension, or ascites, it changes the direction of thinking and raises suspicion of complications that are not externally visible but that are already developing internally [20,76,140].

It is important to emphasize something that is sometimes overlooked in practice, namely, that a complication may occasionally not manifest through one pronounced sign but through a slow, yet unconvincingly normal, recovery. The patient is not eating or getting up as expected, pain lasts longer, and the parameters are not improving. It is precisely such situations that require the greatest clinical maturity [10,34,141]. The skill lies not only in recognizing an obvious catastrophe but in registering an unconvincing deviation before it becomes obvious. On that skill depends whether the complication will be recognized early or late. The main early postoperative red flags, together with their most likely clinical direction and rational next diagnostic step, are summarized in Table 5.

### 7.2. Diagnostic Work-Up

When the postoperative course no longer appears as expected, the diagnostic approach must be purposefully structured rather than improvised. The first step is to differentiate whether the clinical picture most closely resembles bleeding, an intra-abdominal/inflammatory complication, a urinary complication, respiratory deterioration, or a combination of several processes. This initial working orientation is not the final diagnosis, but it helps accelerate the diagnostic process and avoid wasting time on unnecessary steps. Basic laboratory analyses are valuable because they provide rapid orientation. Complete blood count, CRP, and renal function parameters can very quickly indicate whether the clinical picture corresponds to a hemorrhagic or inflammatory complication or is possibly related to compromise of the urinary system [12,16,20,52,76,142].

Abdominal ultrasound retains an important role precisely because it is rapid, available, and suitable for assessing the presence of free fluid. Its strength does not lie in complete characterization of the complication but in the fact that it can quickly confirm that the problem is not merely functional and that it objectively exists. However, when a more precise answer is needed, especially in cases of suspected hemoperitoneum, active bleeding, perforation, or a complex intra-abdominal complication, contrast-enhanced abdominal CT is the diagnostic method that imposes itself. Abdominal CT is the standard for localizing the source of bleeding or perforation, while CT angiography has a special role when there is suspicion of active bleeding that may require an endovascular approach [143,144,145].

In cases of suspected urinary complication, the diagnostic pathway must be equally rapid, but directed differently. Reliance on general laboratory testing alone is not sufficient. When oliguria, elevated creatinine, ascites, persistent pain, urine in the drain, or other elements raising suspicion of injury to the bladder or ureter are present, it is necessary to proceed to targeted urologic evaluation. Early cystoscopy and retrograde urography are important methods for confirmation and treatment planning. This is important precisely because this group of complications can easily remain “between disciplines” if one remains too long at the level of an undefined general assessment [141,146,147,148].

Diagnostic work-up, however, is not merely a sequence of diagnostic tests and procedures. It also includes the timely involvement of other teams. When the clinical picture suggests a vascular problem, active bleeding, urinary injury, or a serious intra-abdominal complication, calling a urologist, vascular surgeon, interventional radiologist, or another specialist is not a sign of uncertainty but an obligation. Thus, a good approach to complications is not only a good choice of diagnostic methods but also the involvement of specialists from other disciplines, who may be crucial both for the final establishment of the diagnosis and for treatment and a favorable outcome [10,11,16,49,149].

In serious postoperative complications, the goal of diagnostics is not perfect academic classification of the event but timely decision-making that reduces morbidity. Sometimes a few well-chosen findings are sufficient to initiate treatment. In other words, the best approach is not necessarily the most detailed one but the fastest path to a clinically useful answer.

### 7.3. Delayed Diagnosis as a Determinant of Morbidity

In the literature on complications of gynecologic laparoscopy, the timing of diagnosis is one of the main determinants of outcome. This is particularly important precisely because the same complication does not carry the same clinical burden in different temporal scenarios. Bleeding brought under control early is not the same as hemorrhage recognized only after the development of hemodynamic instability [12,15,20]. A small ureteral lesion detected rapidly is not the same as urine observed in the drain or a fistula diagnosed days later [12,23,26]. A limited bowel injury repaired intraoperatively does not have the same clinical context as a delayed-recognized perforation with peritonitis and sepsis [34]. In this sense, the timing of recognition is not an additional variable of the complication but its essential determinant.

A complication is rarely transformed from mild to severe simply because it is “biologically aggressive.” Much more often, it worsens because too much time passes between the first sign of deviation and the response. In that interval, secondary morbidity develops: a larger hemoperitoneum, greater contamination, a stronger inflammatory response, the need for resection, more complex reconstruction, a longer hospital stay, more transfusions, higher costs, and a greater risk of permanent damage. Thus, delayed diagnosis actively changes the course of the disease [11,34,63,64].

Because of the smaller incision, shorter hospitalization, and generally easier average postoperative course compared with the postoperative course after open surgery, the team may be psychologically less prepared to accept that a serious complication is nevertheless developing. This is not a theoretical remark, but a real clinical trap. In this sense, a serious approach to laparoscopic safety also implies mental readiness to recognize an inadequate course in time.

Therefore, the quality of postoperative care cannot be measured only by how many complications are reported but also by when they are detected. A center that rarely misses early deterioration and rapidly activates the diagnostic algorithm thereby reduces secondary morbidity. A more comprehensive view of outcome quality measures not only prevention but also the system’s ability to resolve a complication before it escalates. In this sense, early diagnosis is an integral part of good surgical practice.

## 8. Prevention Strategies and Practical Implications

The practical value of complication prevention in gynecologic laparoscopy lies in a series of concrete decisions made at precisely defined stages of the procedure. Some of these decisions precede entry into the operating room, some determine the course of dissection and hemostasis, and some belong to the final steps of the operation, when the level of attention often unjustifiably declines. In addition, prevention also depends on how the work of the center is organized, how younger surgeons are trained, and how outcomes are monitored and analyzed. Precisely for this reason, it is more useful to speak of levels of prevention than of individual recommendations [12,16,20,76]. These preventive strategies, organized according to the phase of application and their practical goal, are summarized in Table 6. The strength of evidence supporting individual preventive measures is not uniform. Accordingly, the recommendations discussed below are interpreted according to the underlying source of evidence. Where formal professional-society guidance is available, it is cited explicitly; where direct comparative evidence is limited, preventive measures are presented as pragmatic risk-reduction strategies supported by observational, technical, or indirect evidence rather than as definitively proven interventions.

### 8.1. Preoperative Optimization

Preoperative optimization should include identification and correction of modifiable risk factors, including anemia, optimization of glycemic control and relevant comorbidities, and individualized planning of venous thromboembolism prophylaxis, consistent with ERAS-oriented guidance for minimally invasive gynecologic surgery [124,125,134,135].

A structured operative plan, including the anticipated entry strategy, dissection requirements, and specimen-extraction approach when relevant, should be established preoperatively [128,134].

When tissue fragmentation or morcellation may be required, preoperative assessment of malignancy risk and planning of the extraction strategy are essential. Morcellation should be avoided when malignancy is known or clinically suspected, while its use in presumed benign disease requires appropriate patient selection and informed consideration of the potential risk of tissue dissemination [112,133].

### 8.2. Intraoperative Prevention

The entry technique should be individualized according to patient characteristics, prior surgery, anticipated adhesions, and operative circumstances because no single entry technique is optimal for every clinical setting [59,127,128,129]. This approach is consistent with SOGC Guideline No. 412 on laparoscopic entry for gynecologic surgery [128]. During dissection, energy devices should be used judiciously, with particular attention to tissue proximity and lateral thermal spread. These principles are supported predominantly by technical reviews and comparative evidence on energy devices rather than by trials specifically designed around complication prevention [61,130,131]. The third pillar of intraoperative prevention is consistent control of hemostasis. This does not mean only stopping visible bleeding but also checking whether the operative field has remained stable and quiet even after repositioning, irrigation, and completion of the main dissection. A brief, formal inspection is not sufficient. Hemostasis must be confirmed as truly definitive, and not merely apparently adequate. A systematic final assessment of hemostasis and structures exposed to difficult dissection, mechanical manipulation, or energy is advisable before completion of the procedure [12,15,16,132]. Although this principle is consistently supported in complication-focused literature, evidence for a specific standardized final-inspection protocol remains limited. During laparoscopic hysterectomy, intraoperative cystoscopy may facilitate immediate recognition of lower urinary tract injury; specific AAGL practice guidance is available for its use in this setting [77,147]. Timely conversion should be regarded as a safety strategy when continued laparoscopy no longer provides adequate visualization, exposure, or control, rather than as a procedural failure [54,55,114,115,116].

### 8.3. Specific Preventive Measures for Rare but Serious Events

In rarer, but clinically highly significant, complications, preventive measures must be even more concrete. For complications related to pneumoperitoneum, this means reasonable adjustment of insufflation pressures, stable trocar positioning, careful monitoring of ventilation parameters, and close cooperation with the anesthesiology team throughout the procedure. Here, prevention does not depend on a single technical maneuver but on whether the physiologic burden of the procedure is kept within limits that are predictable and tolerable [16,149,150].

For the vaginal cuff, the most important prevention lies in the technique of completing the operation itself. It includes proper colpotomy, preservation of the viability of the cuff edges, reliable closure, and clear postoperative instructions for the patient. These measures appear simple precisely because they are basic, but their importance must not be underestimated. The final reconstructive part of the procedure must carry the same weight as the central part of the operation because complications of this segment rarely spare superficiality [15,22,26,90].

For larger trocar sites, particularly those enlarged during specimen extraction, fascial closure should be considered as a risk-reduction measure for port-site hernia [14,97,98,99,151].

In oncologically sensitive situations, technical convenience should remain subordinate to biologic safety. Morcellation should be avoided when malignancy is known or reasonably suspected. When power morcellation is considered appropriate in a carefully selected patient with presumed benign disease, contained tissue extraction should be used in accordance with current professional guidance, while recognizing that containment does not eliminate the possibility of tissue dissemination [112,133,152].

### 8.4. Training, Reporting, and Quality Improvement

The most durable preventive changes do not arise from an individual recommendation but from a system that makes operative behavior more predictable and its quality verifiable. For this reason, structured training, mentorship, and simulation have real preventive value. Their purpose is not only to shorten the learning curve but also to reduce the likelihood that high-risk parts of the procedure will be performed for the first time without sufficient preparation and supervision. In this sense, a good training system actively shapes the process of gaining experience [17,27,85]. Equally important is standardized reporting of complications. The introduction of structured recording of postoperative outcomes, together with standardized registration of intraoperative injuries, enables a center for the first time to see its own results clearly enough to be able to work on them. Without that, there can be no real quality improvement [11,137]. Registries, regular morbidity and mortality meetings, and audits have additional value because they transform a complication from an isolated event into a source of process correction. Their function is not administrative but preventive: they reveal where the protocol was not sufficiently clear, where a decision was made too late, where the standard was breached, and where the same pattern may recur if nothing changes. Only when a complication becomes a reason to change behavior does systemic prevention truly begin to work [15,31].

**Table 6:** Preventive measures in gynecologic laparoscopy: phase of application and practical goals.

Phase	Preventive Measure	Immediate Goal
Preoperative	Correction of anemia and optimization of comorbidities	Increasing the patient’s perioperative reserve
Preoperative	Clear plan for entry, dissection, and extraction	Reducing improvisation during the procedure
Preoperative	Assessment of the acceptability of morcellation/planning of oncologically safe extraction	Prevention of iatrogenic tissue dissemination
Intraoperative	Individualized entry strategy	Reducing the risk of the initial phase of the procedure
Intraoperative	Maintenance of anatomic planes and targeted use of energy	Preserving clarity and technical control
Intraoperative	Active control of hemostasis	Prevention of oversight and early postoperative bleeding
Completion of surgery	Systematic final assessment of the operative field	Detection of problems before leaving the operating room
Completion of surgery	Timely conversion when necessary	Prevention of escalation of a technical problem
Specific events	Consideration of fascial closure for larger or enlarged trocar sites	Prevention of port-site hernia development
Specific events	Careful colpotomy and standardized cuff closure	Reducing the risk of vaginal cuff dehiscence
Specific events	Appropriate patient selection and contained tissue extraction when morcellation is considered	Reducing uncontrolled tissue dissemination while preserving oncologic safety
System level	Structured training and mentorship	Reducing errors associated with inexperience
System level	Standardized reporting, audit, and registries	Transforming complications into a tool for quality improvement

Note: The preventive value of these measures lies not only in their individual effect but also in the fact that together they reduce the margin for error throughout the entire operative course, from preparation to final inspection and system-level learning from outcomes. The evidentiary strength of these measures is not uniform; some are supported by professional-society guidance, whereas others derive primarily from observational studies, technical literature, or indirect evidence and should therefore be interpreted accordingly. The table was synthesized from the studies and guidelines discussed in this section [11,12,14,15,16,17,20,22,26,31,58,76,85,90,108,113,129,130,131,132,133,134,135,137,150,151,152].

## 9. Limits of the Available Evidence and Interpretation of the Literature

The available literature on complications is extensive, but its accumulation does not automatically mean high reliability of conclusions. The greatest problem in this field is not the absence of data but the limited strength of the data that do exist. Most publications allow a reasonable description of complication patterns, recognition of probable risk factors, and formulation of practical precautionary measures. What the literature makes much more difficult is the precise ranking of risks, reliable comparison of different strategies, and drawing strong causal conclusions about which intervention truly changes the outcome and which only appears convincing in the experience of an individual center. In this sense, the limits of the available evidence are not merely a methodological detail; they determine how far this review may go in its claims. The main methodological limitations of the available literature and their practical implications are summarized in Table 7.

### 9.1. Heterogeneity of Definitions

The first and most important limitation is that the literature does not always measure the same thing under the same name. Although this problem was discussed earlier in the paper from the perspective of interpreting studies, its consequence is more important here, namely that when definitions are not aligned, no aggregate estimate can be truly neutral. Some studies reduce a complication to an organ injury requiring repair, others report only major events, others include a broader spectrum of minor postoperative outcomes, and still others use Clavien–Dindo as a therapeutically based framework. Such inconsistency not only makes interpretation of the literature more difficult; it directly limits inference. Because of this, it cannot be stated with certainty that two studies with a similar overall complication rate are truly describing the same clinical burden, nor that two different rates necessarily reflect a real difference in safety. In other words, in this field, a number often has more than one meaning [12,15,18,23,25,27].

### 9.2. Inconsistent Follow-Up and Outcome Capture

The second major limitation arises from the way outcomes are captured over time. Differences in follow-up do not produce only methodological inconsistency, but also change the very structure of observed morbidity. Studies limited to hospitalization systematically favor intraoperative and early clinically obvious complications, whereas papers with active 30-day or longer follow-up also register milder and delayed-manifesting events that would remain invisible under shorter surveillance [12,15,18,20,21]. The consequence is not only a difference in the overall complication rate but also a difference in what is counted at all as the real burden of the procedure. Therefore, published series are not only characterized by different durations of follow-up but in fact present different versions of postoperative reality. This limits any attempt to extract from the literature a single, generally applicable estimate of overall morbidity.

### 9.3. Retrospective Design, Selection Bias, and Limited External Validity

The evidence still derives predominantly from retrospective series, often single-center, and not infrequently based on the experience of a single surgeon or a high-volume team. Such studies have unquestionable value because they provide detailed insight into technique, organization, and complications in a controlled setting. However, the same characteristic that makes them valuable also makes them limited. They do not necessarily represent the reality of the average center. Low complication rates reported in highly specialized institutions cannot be transferred to settings with a lower operative volume, more heterogeneous teams, and a different case structure. Conversely, a higher percentage of complications in registries does not necessarily mean poorer quality of work but rather a more realistic picture of broader clinical practice [17,25,26,27,85]. From this follows an important epistemological limit: the literature shows us relatively well what can be achieved in experienced hands, but speaks less reliably about what is on average to be expected in general practice.

### 9.4. Limited Ability to Evaluate Preventive Measures

One of the most serious shortcomings of the available evidence is that preventive measures, although often reasonably recommended, are much less often rigorously tested. There are relatively few well-designed prospective studies comparing different entry approaches, models of energy management, closure techniques, or tissue extraction procedures with sufficiently strong and clinically relevant outcomes [4,31]. This does not mean that preventive recommendations are not useful. It only means that their evidentiary basis often rests on biomechanical logic, consistent experience, and the indirect support of multiple studies and less often on direct, high-level comparative evidence. A particular problem is that the most severe complications are relatively rare, so studies designed to demonstrate a difference precisely in those outcomes would have to be large, expensive, and logistically demanding. The consequence is that part of the literature on prevention must necessarily rely on less “robust” forms of evidence than would be desirable.

### 9.5. Incomplete Granularity of Published Data

Even when studies report complications correctly, they often lack the clinical granularity needed for deeper interpretation. The exact location of the lesion, the mechanism of injury, its relation to the phase of the operation, the immediate diagnostic pathway, and the precise method of management are not always described clearly enough. This is particularly important in the urinary tract, thermal injuries, complex hemorrhagic events, and complications that manifest later. Without these details, the literature may show that a complication existed, but explains much less well how it occurred and what would most likely have prevented it [15,17,31]. It is precisely at this point that the difference arises between descriptive literature and literature that is truly operationally useful. Registries contribute to this problem. They provide a broader and more realistic epidemiologic picture, but often do not offer an adequate level of technical detail necessary for analyzing the possibilities for prevention.

### 9.6. Rare Complications and the Asymmetry of Evidence

The rarest complications represent a particular methodological challenge. For them, the literature is almost by definition not symmetrical [15,18,21]. What is dramatic, unusual, or oncologically disturbing has a greater likelihood of being published than what was resolved without major consequence. For this reason, port-site metastases, gas embolism, dissemination after morcellation, and similar events carry a high publication intensity relative to their true prevalence. This asymmetry does not make these complications unimportant. On the contrary, they remain clinically significant precisely because of their consequences. However, it does mean that they should be interpreted with particular caution. The literature on them describes possible scenarios and mechanisms better than it allows reliable estimation of their true incidence or precise quantification of absolute risk. This limitation is essential and must not be concealed by stylistic confidence in writing, but needs to be stated clearly.

### 9.7. What These Limitations Mean for Interpretation

Taken together, the limitations of the available literature do not disqualify the existing evidence, but they do change the way it should be used. From it, reliable lines of reasoning can be drawn, for example, which complications predominate in certain operations, which clinical and technical circumstances are consistently repeated as unfavorable, and which preventive decisions have a strong rational basis. What cannot be done with the same level of certainty is to convert that literature into a single table of absolute risks or a strictly hierarchized set of recommendations that would be equally applicable across all centers and for all procedures. In this sense, this review does not pretend to resolve the heterogeneity of the literature; on the contrary, it attempts to make the literature visible and clinically useful.

## 10. Conclusions

Complications of gynecologic laparoscopy cannot be meaningfully interpreted through a single overall incidence rate. Their clinical significance is determined by the interaction between patient- and procedure-related risk, technical mechanisms of injury, surgeon experience, and the timeliness of recognition and response. Hemorrhage, bowel injury, and urinary tract injury remain the principal sources of major morbidity, whereas less frequent complications related to pneumoperitoneum, vaginal cuff failure, port sites, and tissue dissemination may have substantial consequences despite their lower incidence.

Safe laparoscopic practice therefore depends on an integrated pathway that begins with preoperative risk assessment and procedural planning, continues with technically disciplined surgery and early recognition of evolving complications, and extends to structured postoperative surveillance, training, reporting, and audit. Because the available literature is heterogeneous in definitions, follow-up, procedural complexity, and study design, reported rates should be interpreted contextually rather than as directly comparable measures of safety. The practical contribution of this review is to synthesize these heterogeneous data into a clinically oriented framework linking risk architecture, prevention, and early recognition, rather than to propose a single summary complication rate.

## Figures and Tables

**Table 1:** Definitions, classification, and follow-up duration in key studies on complications of gynecologic laparoscopy.

Study	Design/Setting	Population/Type of Procedure	How Complication Was Defined	Classification	Follow-Up Duration	Reported Complication Rate	Key Methodological Note
Bojahr et al., 2006 [17]	Single-center series, standardized technique	1706 LASH procedures	Perioperative events; focus on clinically relevant complications	Intraoperative/postoperative; in practice also within a major/minor framework	Appears to be predominantly perioperative/early postoperative	Postoperative complications around 1.2%; intraoperative rates among the lowest in large series	Standardized single-center technique limits direct comparability with registry data
Johnston et al., 2007 [27]	Prospective single-center series	1265 operative laparoscopies	Focus primarily on major complications	Major complications	Not explicitly stated; emphasis on perioperative/major events	Overall complication rate not reported; conversion due to complications approximately 0.1–0.2%	This type of design captures severe events but probably underestimates minor and delayed complications
Kumakiri et al., 2010 [23]	Single-center high-risk cohort	307 patients with previous laparotomies	Complication defined narrowly as an intraoperative organ injury requiring repair	Organ-oriented/intraoperative	Also records postoperatively diagnosed events; broader than hospitalization alone	Intraoperative organ injuries up to 11.4% in a high-risk population	The study measures a narrower but more severe segment of complications; the population is high-risk and not directly comparable with general cohorts
Donnez et al., 2010 [26]	Single-center series/single-surgeon experience	Laparoscopic hysterectomies for benign indications	Overall and major complications, with emphasis on clinically significant outcomes	Overall/major	Formal follow-up not specified; appears to be predominantly early perioperative evaluation	Around 2% overall, of which approximately 1.5% is major	Single-center, single-surgeon design limits generalizability
Fuentes et al., 2014 [15]	Large single-center series	2888 gynecologic laparoscopic procedures	Broadly defined spectrum of intraoperative and postoperative events	Major/minor	Active recording throughout the entire postoperative course; broader than hospitalization alone	Around 6.2% postoperative complications: 1.93% major and 4.29% minor	Broader outcome capture includes milder events and therefore yields a more comprehensive, but higher, overall complication rate
Sinha et al., 2016 [18]	Single-center retrospective series	448 gynecologic 3D laparoscopic operations	Postoperative events assessed through their therapeutic consequence	Clavien–Dindo	Not specified; appears to be short-term postoperative	Around 2.7% postoperative complications	Use of Clavien–Dindo improves severity classification, although the smaller series and narrower setting limit direct comparison
Kaya et al., 2021 [12]	Large cohort	Gynecologic laparoscopic procedures of varying complexity	Intraoperative and postoperative complications; postoperative complications classified according to therapeutic severity	Clavien–Dindo for postoperative events	6 weeks	Postoperative complications around 6.5%, of which approximately 3.8% were Clavien ≥3; intraoperative complications up to 5.6% in the analyzed series	Broader follow-up captures more delayed and minor events; therefore, the reported rate is not directly comparable with studies limited to hospitalization
Choudhary et al., 2023 [21]	Retrospective single-center series, single-surgeon	Total laparoscopic hysterectomies	Clinically relevant perioperative and postoperative complications	Major/minor framework	Not explicitly specified; appears to be predominantly early postoperative evaluation	Around 3% postoperative complications	The single-surgeon design increases technical homogeneity but reduces external validity
Salmanov et al., 2024 [20]	Multicenter registry	>14,000 laparoscopies for benign indications	Broadly defined spectrum of complications, including minor events	Major/minor	30 days	16.2% overall, of which 5.04% were major and 11.16% minor; 74.9% of complications were detected after discharge	Active 30-day surveillance and broad outcome definitions capture substantially more complications, including post-discharge events
Durdağ et al., 2024 [22]	Single-center series	1515 laparoscopic hysterectomies	Clinically relevant perioperative and postoperative complications	Major/minor framework	Not specified	Around 3.5% postoperative complications; transfusions 11.6% in benign and 13.5% in malignant indications	Differences in indication and procedural composition influence reported complication rates, particularly bleeding and transfusion rates

Note: This table is intended to illustrate why reported complication rates are not directly comparable across studies. The same nominal outcome may represent different levels of morbidity depending on whether a study records only major events, applies the Clavien–Dindo classification, includes minor complications, limits follow-up to hospitalization, or uses active post-discharge surveillance. Accordingly, differences in definitions, classification systems, study populations, and follow-up duration should be considered when interpreting reported complication rates. The table was synthesized from the studies summarized here [12,15,20,23,27,30,32,39,43,44].

**Table 4:** Risk architecture of complications in gynecologic laparoscopy: factors, dominant effects, and practical implications.

Risk Domain	Specific Factor	Dominant Effect on the Operation	Complications Most Affected	Practical Implication
Patient-related	Previous abdominal/pelvic operations	Adhesions, distorted anatomy, more difficult entry	Bowel injury, ureteral injury, vascular injury, conversion	Plan an alternative/safer entry; expect longer and more demanding dissection
Patient-related	Dense adhesions	Loss of dissection planes, poorer visualization	Bowel injury, bleeding, ureteral injury, conversion	Assess the likelihood of adhesions preoperatively; maintain a low threshold for changing strategy
Patient-related	Large uterus/large myomas/masses	Reduced working space, more difficult dissection and extraction	Bleeding, transfusion, longer operation, conversion	Plan the approach and extraction in advance; assess the realistic feasibility of laparoscopy
Patient-related	Obesity	More difficult access, deeper operative field, poorer visualization	Conversion, local port-site complications, longer operative duration	Adapt positioning, port placement, and the expected duration of the procedure
Patient-related	Age/higher ASA/comorbidities	Reduced physiologic reserve	Thromboembolism, infection, respiratory complications, slower recovery	Broader preoperative optimization and more intensive perioperative monitoring
Patient-related	Preoperative anemia/coagulopathy	Reduced tolerance to blood loss	Hemorrhagic complications, transfusion, prolonged recovery	Correction of anemia and hemostatic assessment before surgery
Procedure-related	Procedural complexity	More steps, longer exposure to critical zones	Bleeding, bowel injury, urinary tract injury, conversion	Stratify the procedure by risk, not only by the name of the procedure
Procedure-related	Method of abdominal entry	Determines the safety of the initial phase of the operation	Entry-related vascular and bowel injuries	Individualize the entry strategy according to previous operations and expected adhesions
Procedure-related	Use of energy	Risk of thermal, delayed lesions	Bowel injury, ureteral injury, cuff problems	Limited and thoughtful use of energy in critical zones
Procedure-related	Prolonged operative duration	Combined effect of fatigue, physiologic burden, and cumulative technical compromises	Bleeding, respiratory complications, thromboembolism, conversion	Monitor duration as a sign of increasing operative complexity
Procedure-related	Specimen extraction/final steps	Local stress on the ports and cuff; oncologic risk during manipulation	Port-site hernia, infection, cuff dehiscence, dissemination	Careful closure, ENDOBAG, avoidance of rough enlargement of the incision, and inappropriate morcellation
Surgeon-related	Limited experience	Poorer anticipation and lower tolerance for unexpected anatomy	All major complications, especially conversion and poorly managed complications	Structured training, mentorship, and case selection according to level of experience
Center-related	Low center volume	Less routine and standardization	Greater variability of outcomes and slower escalation when complications occur	Referral of high-risk cases to more experienced centers
Center-related	Low standardization	Greater procedural variability	Bleeding, missed injuries, inconsistent postoperative surveillance	Standard protocols for entry, hemostasis, final inspection, and early diagnosis
Center-related	Poor registration and audit	Underestimation of the true morbidity burden	Especially minor and delayed-recognized complications	Transparent reporting, registries, M&M meetings, 30-day follow-up

Note: The same factor rarely “contributes” to only one complication. Its true significance lies in the fact that it changes the entire course of the operation: visualization, duration, need for energy use, physiologic burden, and the possibility of timely correction of the operative plan. Therefore, risk assessment in gynecologic laparoscopy must be layered, rather than one-dimensional.

**Table 5:** Early postoperative red flags after gynecologic laparoscopy and their most likely clinical direction.

Red Flag/Pattern	What Makes It Suspicious	What Should Be Considered First	Next Rational Step
Pain that worsens instead of subsiding	Does not follow the expected rhythm of recovery; changes in character; accompanied by distension or tachycardia	Hemoperitoneum, bowel injury, urinoma, intra-abdominal infection	Clinical examination + laboratory tests + urgent imaging work-up
Abdominal distension	Increases or is associated with pain, nausea, or ileus	Bleeding, perforation, ileus, urinoma	Ultrasound as the initial assessment, followed by contrast-enhanced CT as indicated
Greater-than-expected drop in hemoglobin	Not proportional to the estimated blood loss; accompanied by tachycardia/weakness	Postoperative bleeding, hemoperitoneum	Laboratory confirmation + ultrasound/CT; consider CT angiography if there is suspicion of active bleeding
Tachycardia without a clear explanation	Persists or worsens despite analgesia and hydration	Bleeding, sepsis, thromboembolism, severe pain-related complication	Assessment of vital signs, laboratory tests, and further targeted work-up
Elevated temperature that persists or rises	Does not appear to be a transient early postoperative response	Infection, abscess, peritonitis, urinary complication	Laboratory tests + clinical assessment of the source + targeted imaging
Oliguria/elevated creatinine	Cannot be explained by hypovolemia or perioperative circumstances	Ureteral or bladder injury, severe systemic stress	Urologic evaluation, cystoscopy/retrograde urography as indicated
Respiratory deterioration	Desaturation, dyspnea, unexpectedly poor respiratory status	Pneumothorax, pulmonary embolism, pneumoperitoneum-related problem	Rapid respiratory and hemodynamic assessment + appropriate imaging
“Unconvincingly” slow recovery without one dominant sign	Several smaller deviations that cumulatively create a poor clinical impression	Early phase of a serious complication that has not yet developed its full picture	Do not rationalize it; repeat the assessment and lower the threshold for diagnostics

Note: No individual sign is, by itself, pathognomonic. Their true value lies in their relationship to the expected postoperative course and in the way they cluster together. It is precisely this pattern of deviation that should trigger targeted diagnostic evaluation. The data were synthesized from the studies and reviews discussed in this section [7,12,15,20,23,31,39,114,115,116,117,118,119,120,121,122,123,124,125,126,127,128,129,130,131,132,133,134,135,136,137].

**Table 7:** Main limitations of the available evidence on complications of gynecologic laparoscopy and their practical significance.

Limitation	How It Manifests in the Literature	What It Means in Practice
Non-uniform definitions of complications	Different studies measure different outcomes under the same name	Overall complication rates are not directly comparable
Different classification of severity	Major/minor, Clavien–Dindo, only organ injuries	It is not always clear whether studies are referring to the same level of morbidity
Inconsistent follow-up	Hospitalization versus 30-day or longer surveillance	Part of the postoperative burden remains invisible in shorter follow-up
Dominance of retrospective single-center series	Experiences of high-volume centers and individual operators	The results have limited generalizability to other centers
Limited number of prospective comparative studies	Preventive measures are rarely tested in a robust design	Recommendations are often rational, but not definitively evidence-based
Insufficient clinical granularity	Poorly described mechanisms, lesion location, and course of management	It is more difficult to link a technical decision to a specific outcome
Asymmetrical literature for rare complications	Cases with greater clinical impact are more often published	Possible scenarios are well described, but absolute risk remains uncertain
Insufficient standardization of reporting	Variable quality and depth of reported data	Limited ability to compare centers and strategies precisely

Note: These limitations do not mean that the literature on complications of gynecologic laparoscopy is clinically unusable. They mean that it is most useful when used to identify patterns, rather than for falsely precise summation of non-comparable figures. The table was synthesized from the studies discussed in this section [12,15,17,18,20,21,23,25,26,27,31,85].

## Data Availability

Data presented in this study are available from the corresponding author upon request.

## Abbreviations

The following abbreviations are used in this manuscript:

ASA	American Society of Anesthesiologists
BMI	Body Mass Index
CT	Computed Tomography
CTA	Computed Tomographic Angiography
CRP	C-reactive Protein
Hb	Hemoglobin
SpO_2_	Peripheral Oxygen Saturation
PIP	Peak Inspiratory Pressure
